# Chromosome-level genome assembly and annotation of the gynogenetic large-scale loach (*Paramisgurnus dabryanus*)

**DOI:** 10.1038/s41597-025-04498-8

**Published:** 2025-01-26

**Authors:** Lei Zhang, Wanting Zhang, Yingyin Cheng, Yutong Fang, Xin Guan, Ao Gong, Yanxin Jiang, You Duan, Lei Huang, Waqar Younas, Yaping Wang, Mijuan Shi, Xiao-Qin Xia

**Affiliations:** 1 Key Laboratory of Breeding Biotechnology and Sustainable Aquaculture (CAS), Hubei Hongshan Laboratory, Key Laboratory of Aquaculture Disease Control, Ministry of Agriculture and Rural Affairs, The Innovation Academy of Seed Design, Institute of Hydrobiology, Chinese Academy of Sciences, Wuhan, 430072 China; 2 College of Advanced Agricultural Sciences, University of Chinese Academy of Sciences, Beijing, China; 3 College of Fisheries and Life Science, Dalian Ocean University, Dalian, 116023 China; 4 Hefei Bestspectra Medical Lab Co., Ltd., 2800 Chuangxin Road, Hefei, 230000 P. R. China; 5 The Joint Laboratory for Lung Development and Related Diseases of West China Second University Hospital, Sichuan University and School of Life Sciences of Fudan University, West China Institute of Women and Children’s Health, West China Second University Hospital, Sichuan University, Chengdu, China; 6 NHC Key Laboratory of Chronobiology (Sichuan University), Chengdu, China; 7 Yancheng Agricultural College, Yan Cheng, 224051 China

**Keywords:** Genome, Genomics

## Abstract

The large-scale loach (*Paramisgurnus dabryanus*; Cypriniformes: Cobitidae) is primarily distributed in East Asia. It is an important economic fish species characterized by fast growth, temperature-dependent sex determination and the ability to breathe air. Currently, molecular mechanism studies related to some aspects such as sex determination, toxicology, feed nutrition, growth and genetic evolution have been conducted. However, the lack of a high-quality reference genome has hindered further research. In this study, we performed PacBio HiFi and Hi-C sequencing on a female (ZW) specimen and assembled the first high-quality chromosome-level genome of the large-scale loach. The assembled genome comprises 24 chromosomes, with a total length of 1.04 Gb, a scaffold N50 of 41.7 Mb, and a BUSCO completeness of 95.8%, including 28,311 protein-coding genes. These findings not only provide new insights into the genome structure of the large-scale loach but also establish a crucial reference point for omics studies and serve as an essential genomic resource for breeding programs in this species.

## Background & Summary

The large-scale loach (*Paramisgurnus dabryanus*), belonging to the order Cypriniformes, family Cobitidae, and subfamily Cobitinae, is a small, benthic freshwater fish primarily distributed in East Asia. Highly enriched with protein, fats, minerals, and various vitamins, it is considered one of the most important aquaculture species due to its high nutritive and economical values^1–3^.

Karyotype analysis has shown that the sex chromosomes of large-scale loach follow a ZZ/ZW system, with females being heterogametic (ZW)^4,5^. This species exhibits sexual dimorphism in growth phenotypes, with females being larger and growing faster than males. Gonadal differentiation occurs 30 days post-fertilization in males and 45 days in females, with sexual maturity reached within six months. In addition to genotypic factors, temperature plays a crucial role in the sex determination of large-scale loach. Studies have shown that at 20 °C, the sex ratio remains 1:1, but at 25 °C, the proportion of males exceeds 70%, with this ratio gradually increasing as the temperature rises further, making large-scale loach an ideal model for studying temperature-dependent sex regulation^6,7^. Current research on sex determination focuses on the development of sex markers and the molecular mechanisms of temperature’s effect on sex determination. However, the lack of an available reference genome has hindered the identification of universal sex-specific molecular markers^8^, and research on the impact of temperature on sex determination remains in its early stages^9,10^. Although the mitochondrial genome is frequently employed in phylogenetic analysis^11^, evolutionary genomics^12^, and species identification^13^ of large-scale loach, its restricted genetic renders it unsuitable for the investigation of any economic trait. Assembling a chromosome-level reference genome of large-scale loach will not only aid in the detailed study of chromosomal structure but also provide a foundation for investigating the mechanisms underlying sex determination in this species.

In recent years, various omics technologies have played an important role in unraveling the complex molecular and genetic mechanisms of large-scale loach. However, due to the lack of a reference genome, researchers have often relied on *de novo* strategies, which focus on only a limited number of genes and cannot fully explore related mechanisms or reveal the species’ complex biological characteristics. For example, *de novo* transcriptomics has been used to explore the ammonia tolerance and detoxification mechanisms of this species^14–16^, identifying key regulatory genes that may be used in fish breeding^17^. Additionally, combined metabolomics and *de novo* transcriptomics analyses have preliminarily examined the effects of different protein and lipid ratios on growth performance and their molecular mechanisms^18^. Without genome support, these studies have been unable to fully elucidate the regulatory mechanisms involved. Furthermore, omics tools have been increasingly applied in studies related to growth^1,19^, pathogen infection and prevention^20^, environmental toxicology^21–23^, and interspecies hybridization^24,25^ of large-scale loach.

The publication of the genome will significantly advance omics studies of large-scale loach, providing a more comprehensive genetic background for identifying new gene functions and biological pathways, and contributing to future studies on systematics, evolutionary analysis, and genetic breeding.

In this study, we employed PacBio sequencing and Hi-C assembly technologies to construct a chromosome-level reference genome for large-scale loach. Based on the karyotype (2n = 48), 99.56% of the assembled contig sequences were successfully anchored to 24 chromosomes. The total genome assembly length was 1.04 Gb, with a scaffold N50 of 41.7 Mb and a BUSCO completeness score of 95.8%, containing 28,311 protein-coding genes, 97.49% of which were annotated. This genome provides crucial data for the development of molecular markers related to economically important traits such as growth and sex-specific traits, as well as a foundation for identifying key genes and elucidating molecular mechanisms.

## Methods

### Ethics statement

All experiments and animal treatments were carried out according to the principles of the Animal Care and Use Committee of the Institute of Hydrobiology, Chinese Academy of Sciences.

### Sample collection and gynogenesis

**Fig. 1:**
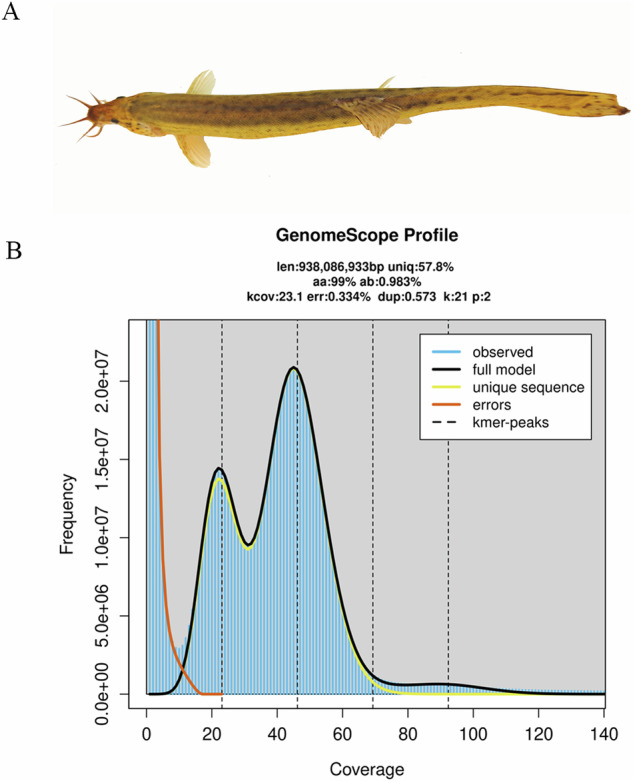
Genome survey and morphological characterization of gynogenetic offspring of the large-scale loach for genome assembly. (**A**) Dorsal view of the gynogenetic offspring of the large-scale loach. (**B**) The frequency distribution of 21-kmer depths.

A set of large-scale loaches was sourced from the Baishazhou Aquatic Product Market in Wuhan, Hubei Province, China. Jellyfish (v2.2.10)^26^ and GenomeScope (v2.0)^27^ were employed to analyze the frequency distribution of 21-kmer depths, estimating the average heterozygosity of the large-scale loach genome via ten randomly selected female samples. A sexually mature female with a heterozygosity of 1.44% was selected as the parent for gynogenetic induction through heat shock. This method inhibited the extrusion of the second polar body, resulting in gynogenetic progeny with lower heterozygosity. One female individual from the progeny was subsequently selected for further analysis (Fig. 1A). After anesthetization with MS222 (0.05%), blood and muscle tissue samples were immediately collected, flash-frozen in liquid nitrogen and stored at −80 °C for future analysis.

### Illumina sequencing and genome survey

Genomic DNA was extracted from both the gynogenetic parent and offspring using a modified cetyltrimethyl ammonium bromide (CTAB) protocol. Whole-genome resequencing was conducted using the paired-end (PE-150) strategy on the Illumina DNBSEQ-T7 platform. Additionally, whole-genome sequence data from ten female large-scale loaches from our previous study^28^ were incorporated for comparative genome survey analysis. GenomeScope (v2.0) was employed to analyze the data from the gynogenetic parent, offspring, and the ten female loaches. The results of the resequencing and genome survey analyses indicated a notable reduction in heterozygosity to 0.98% in the gynogenetic offspring, a figure that is significantly lower than that observed in the parent (1.44%) and the wild population (1.69%) (Table 1). The genome size of the large-scale loach was estimated at 0.94 Gb, based on 59.42 GB of clean sequencing data, closely aligning with the 1.04 Gb size obtained from the assembly (Fig. 1B).

**Table 1:** The estimated genome sizes and heterozygosities of the gynogenetic offspring, the female parent, and ten other females.

Sample	Heterozygosity (%)	Lengh of Haploid Genome (Gb)	Clean data (GB)
The gynogenetic offspring	0.98	0.94	59.42
The female parent	1.44	0.95	33.55
The wild population			
Female #1	1.81	0.96	25.99
Female #2	1.43	0.97	27.17
Female #3	1.74	0.96	28.44
Female #4	1.89	0.97	22.17
Female #5	1.83	0.95	25.05
Female #6	1.83	0.96	24.10
Female #7	1.77	0.94	29.72
Female #8	1.40	0.93	33.48
Female #9	1.81	0.95	27.87
Female #10	1.37	0.98	24.44
Average	1.69	0.96	26.84

### Hi-C based and PacBio sequencing

Hi-C libraries were constructed following established protocols^29^. In brief, samples were cross-linked with 1% formaldehyde for 10 minutes at room temperature, then cool down with 0.125 M glycine for 5 minutes. The cross-linked cells were lysed and endogenous nucleases were inactivated with 0.3% SDS. Chromatin DNA was digested with 100U MboI (NEB), labeled with biotin-14-dCTP (Invitrogen), and ligated using 50U T4 DNA ligase (NEB). After reversing the cross-links, the ligated DNA was extracted using the QIAamp DNA Mini Kit (Qiagen) following the manufacturer’s instructions. The purified DNA was cut into 300–500 bp fragments, blunt-end repaired, A-tailed, and adaptors were added. Biotin-streptavidin pull-down followed by PCR amplification was then performed. The Hi-C libraries were quantified and sequenced on the MGISEQ-2000 platform (BGI, China).

For PacBio sequencing, a genomic library with inserts ranging from approximately 15 K to 20 K bp was prepared using the SMRTbell Express Template Prep Kit 3.0 (Pacific Biosciences, Menlo Park, CA, USA). Sequencing was conducted on the PacBio Sequel II platform (Pacific Biosciences, Menlo Park, CA, USA), following the manufacturer’s protocol. To process raw sequencing reads, default parameters of min passes = 3 and min RQ = 0.99 in the CCS software (https://github.com/PacificBiosciences/ccs) were applied to generate high-fidelity (HiFi) reads with quality exceeding Q20. After quality control and filtering, the final dataset comprised 65.55 GB of high-quality long reads (Table 2).

**Table 2:** Statistics of sequencing data.

Library type	Sample	Platform	Clean data (GB)	Read N50 (bp)
Illumina sequencing	Tail	Illumina DNBSEQ-T7	59.42	150
PacBio sequencing	Tail	PacBio Sequel II	65.55	14,598
Hi-C	Blood	BGI MGISEQ-2000	121.75	150
RNA-seq sequencing	Gonad	Illumina NOVASEQ 6000	123.78	150

### Genome assembly

We used HiFi data for contig assembly with hifiasm^30^ (v 0.15.2, parameters: --h1 --h2 -s 1 -10), resulting in haplotype A and haplotype B. Haplotype A assembly contained 1.05 Gb in 501 contigs with a contig N50 of 40.57 Mb, while haplotype B assembly contained 1.04 Gb in 374 contigs with a contig N50 of 45.64 Mb. Both haplotypes were scaffolded using Lachesis^31^ based on valid Hi-C data processed via HiC-Pro^32^ (v2.9.0), producing two chromosome-level haploid genomes, Haplotype A and Haplotype B. Haplotype B had a slightly higher mapping rate of short reads from second-generation sequencing compared to Haplotype A (Supplementary Table S1) and demonstrated higher completeness, making it the reference genome used for structural and functional annotation. Based on karyotype analysis, the assembled contigs were anchored to 24 chromosomes with a length of 1,038,659,332 bp, representing 99.56% of the total genome length. The final genome assembly was 1,043,469,091 bp in length, comprising 219 contigs, including both chromosomes and unanchored fragments (Table 3).

**Table 3:** Statistics of genome assemblies of large-scale loach, pond loach (*Misgurnus anguillicaudatus*) and a species of plateau loaches (*Triplophysa dalaica*).

	Large-scale loach	Pond loach	Plateau loach
Karyotype	ZZ/ZW	XX/XY	—
No. of scaffolds	219	357	141
No. of chromosome-level scaffolds	24	25	25
Scaffolds N50 (Mb)	41.7	43.0	23.6
Scaffolds max length (Mb)	76.67	17.20	36.52
Total size (Gb)	1.04	1.10	0.61

### Repeat sequence annotation

We identified repeat sequences and transposable elements (TEs) using the methods of *de novo* assembly^33^ and homologous prediction. First, we used RepeatModeler^34^ (v2.0.2) to predict the repeat sequence with default parameters. Then, RepBase database^35^ and RepeatMasker^36^ (v4.1.2) were used to annotate the sequence homologs. The results showed that 615.59 Mb are repeat sequences, accounting for 58.99% of the large-scale loach genome. Among these repeat sequences, most (37.74%) are DNA transposons, followed by 8.72% of unclassified elements and 3.86% of long terminal repeats (LTRs) (Table 4).

**Table 4:** Classification statistics of repeated sequences in the genome of large-scale loach.

Repeat Classes		Number of Elements	Length (bp)	Percentage of genome (%)
Retro elements	1. SINEs	43,731	6,592,529	0.63
	2. LINEs	87,924	21,417,910	2.05
	3. LTR elements	83,079	40,285,557	3.86
	Total Retro elements	203,734	68,317,130	6.55
DNA transposons	—	1,825,308	393,796,610	37.74
Rolling-circles	—	39,648	4,199,431	0.4
Unclassified	—	299,643	91,009,449	8.72
Total interspersed repeats	—	1,568,085	553,713,189	53.07
Small RNA	—	22,410	5,334,963	0.51
Satellites	—	26,238	9,159,354	0.88

### Coding gene prediction

We used three complementary approaches for coding gene prediction: (1) *de novo* prediction, homology-based prediction, and transcriptome-based prediction. *De novo* prediction: Augustus (v3.1.0, parameters: --species=zebrafish --gff3=on --strand=both)^37^, SnapGene (v2006-07-28)^38^, GeneID (v1.4), GlimmerHMM (v3.0.1), and GeneMarkES (v4.48)^39^ were employed. (2) Homology-based prediction: GeMoMa (v1.9)^40^ was used to predict homologous genes from zebrafish, common carp, and loach species. (3) Transcriptome-based prediction: We collected publicly available transcriptomic data (PRJNA266739^41^ and PRJNA623189^19^) along with self-generated data. Two assembly methods were employed: reference-guided assembly using Hisat2 (v2.1.0)^42^ and StringTie (v2.1.4)^43^, and *de novo* assembly using Trinity 40 (v2.1.1)^44^, followed by PASA (v2.4.1)^45^ to align the assembled transcripts back to the reference genome for gene prediction.

**Fig. 2:**
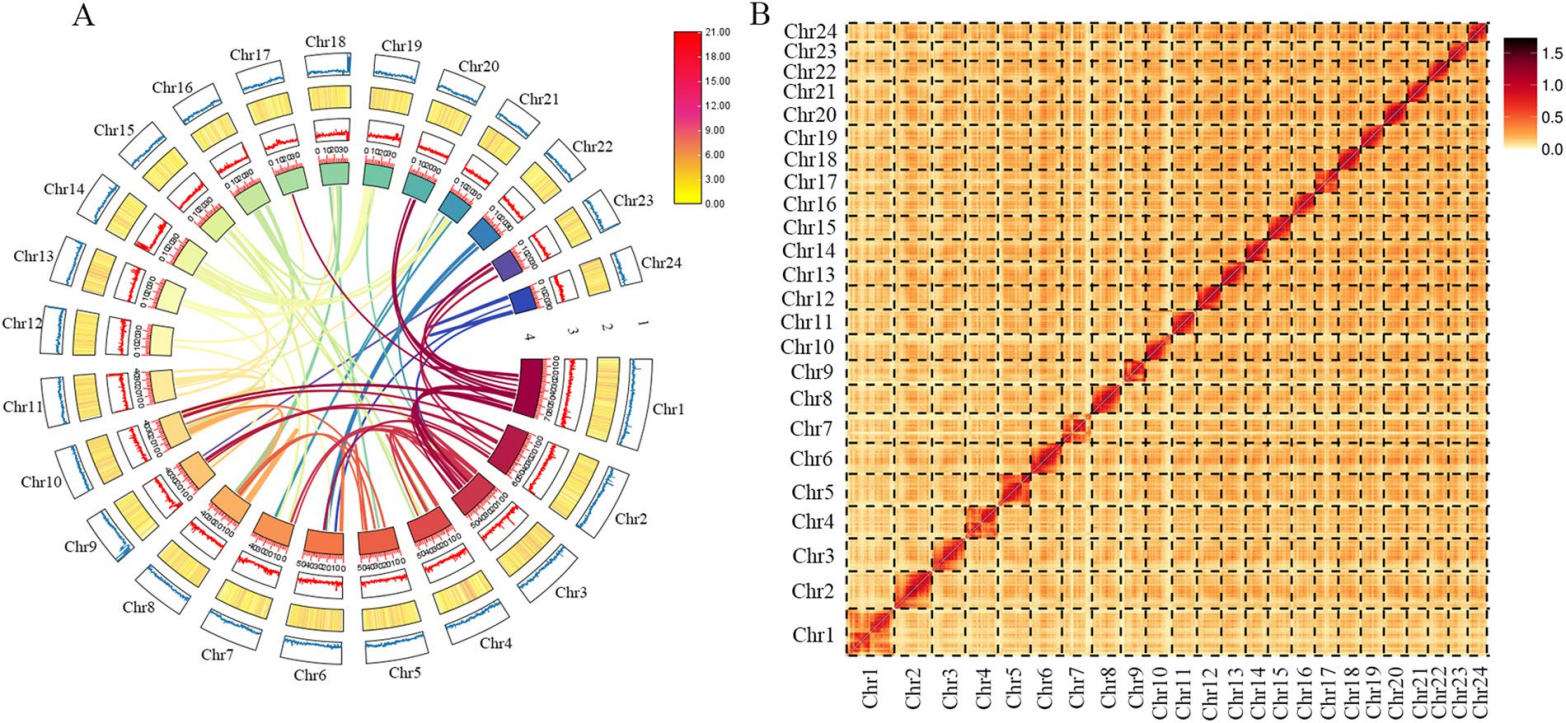
The profiles of the genome structure and interactions among genes. (**A**) The circos plot showing the features of the large-scale loach genome. Tracks from outer to inner layers represent the 24 chromosomes, repeat elements density, gene density, GC content, and links of intragenomic syntenic blocks within 100 K bp sliding windows. (**B**) Hi-C interaction heatmap indicating the interactions among chromosomes.

Finally, we integrated the predictions from these three methods using EVM (v1.1.1)^46^ and refined them with PASA, resulting in the identification of 28,311 genes (Table 5). Based on the annotation results, syntenic blocks across the 24 chromosomes were identified using MCScanX (https://github.com/wyp1125/MCScanx) with parameters set to -e 1e-10 and -s 5. A circular plot illustrating the distribution of gene and repeat density, GC content, and synteny within the genome was generated using TBtools-II^47^ (Fig. 2A).

**Table 5:** Statistical analyses (average) of the gene structure of large-scale loach and pond loach genome.

Species	Gene number	Gene length (bp)	CDS length (bp)	Exon per gene	Exon length (bp)	Intro length (bp)
Large-scale loach	28,311	15,488.2	2,101.39	11.87	275.19	1,634.05
Pond loach	24,974	22,413.7	1,678.34	9.96	259.43	2,213.89

### Noncoding RNAs annotation

Non-coding RNAs, including tRNAs, rRNAs, miRNAs, snoRNAs, and snRNAs, were annotated using established tools. tRNAs were identified based on structural features using tRNAscan-SE (v2.0.12)^48^ with default parameters. rRNAs were predicted using RNAmmer (v1.2)^49^ (parameters: -S euk -m tsu, lsu, ssu) based on structural characteristics. miRNAs, snRNAs, and snoRNAs were predicted using covariance models from Rfam (v14.1)^50^ through INFERNAL (v1.1.4, parameters: -cut_ga -rfam -nohmmonly -fmt 6)^51^. In total, 2,660 miRNAs, 23,781 tRNAs, 10,126 rRNAs, 1,457 snRNAs, and 387 snoRNAs were annotated (Table 6).

**Table 6:** Annotation of non-coding RNA genes in large-scale loach genome.

Type		Copy Number	Average Length (bp)	Total Length (bp)	Percentage (%)
rRNA	18S	24	1,834.72	440,334	0.0422
	28S	224	4,433.68	993,145	0.0952
	5.8S	240	153.59	36,861	0.00353
	5S	9,638	118.98	1,146,770	0.110
	Total rRNA	10,126	1,635.24	2,617,110	0.251
snRNA	spliceosomal	1,435	159.70	229,171	0.0220
	scaRNA	13	215.85	2,806	0.000269
	other	9	64.22	578	0.0000554
	Total sncRNA	1,457	146.59	232,555	0.0223
snoRNA	—	385	160.09	61,635	0.00591
miRNA	—	2,660	88.25	234736	0.0225
tRNA	—	23,781	77.25	1837114	0.176

### Gene function annotation

Gene functions were annotated through comparisons with public databases, including UniProt (Swiss-Prot and TrEMBL), Pfam (The Protein Families Database), NR (Non-Redundant Protein Database), EggNOG-mapper, KEGG (Kyoto Encyclopedia of Genes and Genomes), KOG (Eukaryotic Orthologous Groups), and GO (Gene Ontology). Diamond (v2.1.8.162)^52^ was used to align the protein sequences predicted by EvidenceModeler with these databases, using an E-value cutoff of 1e−05. The best hits were retained and results from the seven databases were integrated. A total of 27,600 genes (97.49% of the 28,311 predicted protein-coding genes) were annotated, with 97.15%, 84.27%, 81.22%, 88.22%, 84.02%, 18.67%, and 20.23% of genes found in NR, UniProt, Pfam, EggNOG, KOG, KEGG, and GO, respectively (Table 7).

**Table 7:** The number and ratio of genes annotated on various databases.

	KOG	Pfam	UniProt	EggNOG	Nr	GO	KEGG	All
Number	23,786	22,994	23,858	24,975	27,504	5,726	5,285	27,600
Ratio (%)	84.02	81.22	84.27	88.22	97.15	20.23	18.67	97.49

### Chromosomal synteny analysis

**Fig. 3:**
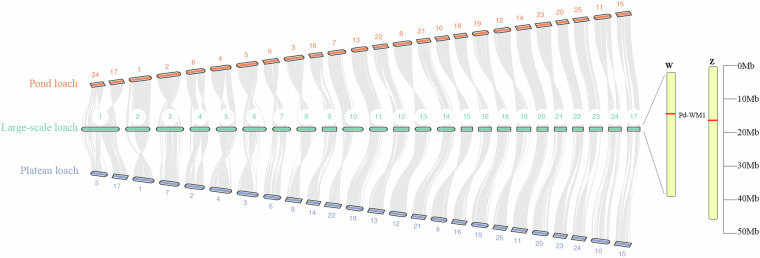
Genome Synteny among Large-scale loach, Pond loach, and Plateau loach. The 17th chromosome, hypothesized to be the sex chromosome, is displayed as the final chromosome in the figure. The female-specific molecular marker Pd-WM1, located on the putative Z and W chromosomes, is shown on the right.

To accurately evaluate genome structural features and validate assembly quality, we performed synteny analysis between the large-scale loach and two other loach species, the pond loach and plateau loach, both of which have available chromosome-level genomes. Using Last (v1559)^53^ and JCVI (v1.3.8)^54^, we identified syntenic gene pairs and homologous regions between each species’ genomes and visualized the results. The analysis revealed significant synteny among the three species’ genomes, with a high degree of chromosomal structural consistency between large-scale loach and both pond loach and plateau loach, further validating the quality of our genome assembly and annotation (Fig. 3).

We used GeMoMa (v1.9) to align homologous genes between Haplotype A and Haplotype B, using Haplotype B as the reference. The two haplotypes exhibited good synteny (Supplementary Figure S1). In our previous studies, we identified a 383 bp sex-specific molecular marker (Pd-WM1) present only in females (NCBI accession no. PQ346371). Using primers designed from this sequence, we observed that in addition to the 383 bp band in females, both males and females exhibited a 544 bp band. Sequence alignment revealed that the longer band contained an insertion compared to the shorter band. BLAST alignment localized the two sequences uniquely to chromosome 17 of Haplotype A and Haplotype B, respectively, suggesting that this chromosome is the sex chromosome. Given the good synteny between the two chromosomes, we designated chromosome 17 of Haplotype B as the W chromosome and that of Haplotype A as the Z chromosome, consistent with the marker length characteristics (Fig. 3).

## Data Records

Raw llumina short read, PacBio long read, and Hi-C sequencing data for generating genome assembly of the gynogenetic offspring and raw llumina short read of the gynogenetic female parent have been deposited in the Genome Sequence Archive (Genomics, Proteomics & Bioinformatics 2021) in National Genomics Data Center (Nucleic Acids Res 2024), China National Center for Bioinformation / Beijing Institute of Genomics, Chinese Academy of Sciences^55,56^ under the accession number CRA019305^57^. RNA-seq data for annotating the large-scale loach assembly are available at Genome Sequence Archive (GSA) of NGDC the accession number CRA018897^58^. The chromosome-level genome assembly, based on Haplotype B, is available in NCBI under accession number GCA_030506205.2^59^. Genome annotation files and Haplotype A assembly results have been deposited in the Figshare database^60^. All files related to assembly and annotation are also available at Aquatic Bioinformatics Archive^61^ .

## Technical Validation

### Quality evaluation of the genome assembly and annotation

The genome was evaluated for quality by mapping second-generation sequencing reads to the assembly using Bowtie2 (v2.1.0)^62^, yielding a mapping rate of 97.16%. For chromosome-level quality assessment, the Hi-C heatmap revealed strong interaction signals along the diagonal without significant noise in other regions (Fig. 2B), indicating high accuracy in chromosome assembly. Genome completeness was evaluated using BUSCO (v5.2.2, parameters: -c 10 -long -f)^63^ with the actinopterygii_odb10 database. The genome showed a BUSCO completeness score of 95.8%, including 94.2% single-copy and 1.6% duplicated genes (Table 8), which is slightly higher than those of the other two loach species, confirming the high completeness of our assembled genome.

**Table 8:** Completeness and accuracy evaluation of the genomes of large-scale loach, pond loach and the plateau loach, *Triplophysa dalaica*.

Type	Large-scale loach	Pond loach	Plateau loach
Complete BUSCOs (C)	3,486 (95.8%)	4,325 (94.35%)	4,294 (93.7%)
Single-Copy BUSCOs (S)	3,429 (94.2%)	3,841 (83.79%)	4,130 (90.1%)
Duplicated BUSCOs (D)	57 (1.6%)	484 (10.56%)	164 (3.6%)
Fragmented BUSCOs (F)	24 (0.7%)	64 (1.40%)	120 (2.6%)
Missing BUSCOs (M)	130 (3.5%)	195 (4.25%)	170 (3.7%)
Total BUSCOs	3,640 (100%)	4,903 (100%)	4,584 (100%)

## Supplementary information

Supplementary information

## Data Availability

All commands and pipelines used for data processing followed the official manuals and protocols of the respective bioinformatics tools. No custom scripts or code were used. Specific parameters for each tool are provided in the Methods section, and default parameters were applied where not specified.
