# Supplementary material for: Chromosome-level genome assembly and annotation of the gynogenetic large-scale loach (Paramisgurnus dabryanus)
Source: Sci Data. 2025 Jan 26;12:155. doi: 10.1038/s41597-025-04498-8 (PMC11770070; doi:10.1038/s41597-025-04498-8)
Supplement: Supplementary file 1 — Supplementary information [file 41597_2025_4498_MOESM1_ESM.docx]

**Table S1**. Bowtie2 Alignment Rate of HaplotypA and HaplotypB.

|  | **Overall alignment rate** | **Unique alignments number (rate)** | **Multiple alignments number (rate)** | **PE unmapping** | **SE alignments** | **Aligned discordantly 1 time** | **Mates** | **Aligned 0 times** |
| --- | --- | --- | --- | --- | --- | --- | --- | --- |
| HapA | 97.11% | 119131798 (60.14%) | 63438481 (32.03%) | 15513226 | 2955903 | 25112846 | 11460830 (45.64%) | 5294251 (21.08%) |
| HapB | 97.15% | 119243204 (60.20%) | 63445698 (32.03%) | 15393703 | 2951523 | 24884360 | 11296989 (45.40%) | 5276491 (21.20%) |


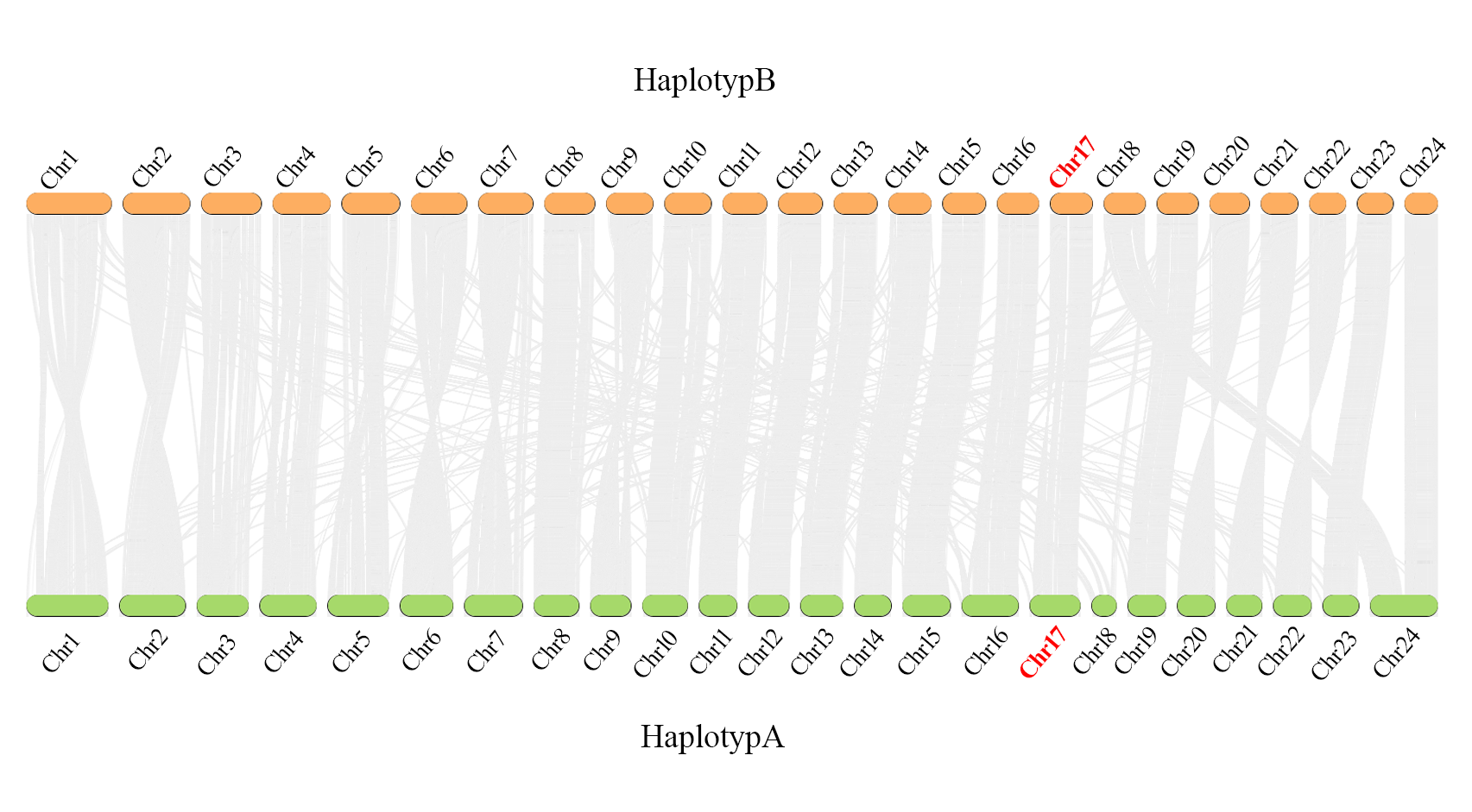


**Figure S1**. Haplotype A and Haplotype B chromosome-level genome collinearity analysis.
